# Investigation of the Phytochemical Composition, Antioxidant Activity, and Methylglyoxal Trapping Effect of *Galega officinalis* L. Herb In Vitro

**DOI:** 10.3390/molecules25245810

**Published:** 2020-12-09

**Authors:** Katarzyna Bednarska, Piotr Kuś, Izabela Fecka

**Affiliations:** Department of Pharmacognosy and Herbal Medicines, Faculty of Pharmacy, Wroclaw Medical University, ul. Borowska 211, 50-556 Wroclaw, Poland; piotr.kus@umed.wroc.pl (P.K.); izabela.fecka@umed.wroc.pl (I.F.)

**Keywords:** galega, guanidines, flavonoids, phenolic acids, polyphenols, methylglyoxal trapping, antioxidant activity

## Abstract

*Galega officinalis* L. has been known for centuries as an herbal medicine used to alleviate the symptoms of diabetes, but its comprehensive chemical composition and pharmacological activity are still insufficiently known. The current study involved the qualitative and quantitative phytochemical analysis and in vitro evaluation of the antioxidative and methylglyoxal (MGO) trapping properties of galega herb. Ultra high-performance liquid chromatography coupled with both the electrospray ionization mass spectrometer and diode-array detector (UHPLC-ESI-MS and UHPLC-DAD) were used to investigate the composition and evaluate the anti-MGO capability of extracts and their components. Hot water and aqueous methanol extracts, as well as individual compounds representing phytochemical groups, were also assessed for antioxidant activity using DPPH (2,2-diphenyl-1-(2,4,6-trinitrophenyl)hydrazyl) and ABTS (2,2′-azino-bis(3-ethylbenz-thiazoline-6-sulfonic acid) assays. Quercetin and metformin were used as a positive control. We confirmed the presence of tricyclic quinazoline alkaloids, guanidines, flavonoids, and hydroxycinnamic acids (HCAs) in galega extracts. The polyphenolic fraction was dominated by mono-, di-, and triglycosylated flavonols, as well as monocaffeoylhexaric acids. The in vitro tests indicated which *G. officinalis* components exhibit beneficial antioxidative and MGO trapping effects. For galega extracts, flavonols, and HCAs, a potent antiradical activity was observed. The ability to trap MGO was noted for guanidines and flavonoids, whereas HCA esters and quinazoline alkaloids were ineffective. The formation of mono-MGO adducts of galegine, hydroxygalegine, and rutin in the examined water infusion was observed.

## 1. Introduction

Before the development of pharmacological anti-diabetic therapy, traditional medicine was widely used to reduce the symptoms associated with type 2 diabetes (T2D) [1]. Among the over 1000 species of plants used as anti-diabetic agents, *Galega officinalis* L. (*Fabaceae*), also known as galega or goat’s rue, deserves some special attention [2]. The flowering aerial parts of galega (*Galegae herba*) were used in the past to alleviate the polyuria associated with long-term hyperglycemia, but also to treat many other conditions from tuberculosis, bubonic plague, and malignant fevers to epilepsy, helminthiasis, and various infectious diseases [3,4]. Moreover, due to its presumed impact on increasing milk yield, galega was used as a galactagogue in humans [5]. At the beginning of the 19th century, it was extensively cultivated as a forage crop in the United States, but in 1986 Keeler et al. [6] reported the clinical symptoms of poisoning in sheep, occurring at doses of about 0.8 g of dried *G. officinalis* herb per kilogram of body weight per day; thus, its continued use in agriculture has been limited [7]. Despite such toxic potential in high doses, *G. officinalis* is still used to manage the early stages of T2D or as part of its complementary treatment in several countries, including Bulgaria and the United Kingdom, where it is recommended for use in diabetes mellitus by the British Medicine Herbal Association [8,9]. The therapeutic dose of galega herb in humans recommended by Youngken in *A Textbook of Pharmacognosy* [10] is 4 g per day; therefore, it is many times lower than the dose causing toxic effects on animals. Early pharmacological studies showed that *Galegae herba* demonstrates hypoglycemic activity and the glucose-lowering effect has been attributed to the presence of guanidine derivatives, especially galegine and hydroxygalegine, in the raw material [11,12]. Galegine became the basis for the synthesis of metformin (1,1-dimethylbiguanide), commonly used as a first-line drug for monotherapy and combination therapy to manage hyperglycemia in T2D [13]. Research on galegine and its derivatives (guanidines and biguanides) was considered a milestone in the development of oral antidiabetic pharmacotherapy. Other than the ability to reduce hepatic gluconeogenesis, increase insulin sensitivity, and inhibit the absorption of glucose, metformin has also been found to be potentially useful in reducing the risk of diabetic vascular complications [14,15]—in particular, those associated with the accompanying increase in reactive carbonyl species (RCS) plasma levels and both their direct tissue toxicity and indirect toxicity by leading to the formation of harmful advanced glycation and oxidation end products (AGEs and AOPPs, respectively) [15]. Currently, among RCS, methylglyoxal (MGO) is considered as the main precursor of the non-enzymatic glycation and oxidation of proteins, which results in the formation of AGEs and AOPPs [16]. One of the possible mechanisms of action responsible for this preventive effect is lowering the RCS concentration by trapping reactions with specific compounds. Several in vitro and in vivo studies have demonstrated that a reduction in the MGO plasma concentration could be an effective strategy for the direct alleviation of diabetic vascular complications [16,17,18]. So far, the established biological properties of *G. officinalis* are anti-hyperglycemic [17], antimicrobial [18], and anti-aggregate [19,20]. However, the literature provides little information on the antioxidant properties, and no research has referred to the methylglyoxal trapping capacity of goat’s rue extracts. Both of these biological activities may contribute to limiting the damage caused by hyperglycemia in complications of diabetes. According to recent research, *Galegae herba* extracts could be beneficial for the prevention of kidney tissue damage in diabetes [21]. Due to the structural similarity to metformin, the galegine present in *G. officinalis* may demonstrate analogy RCS trapping activity. Moreover, many recent studies have reported that other groups of phytochemicals commonly occurring in plants—e.g., flavonoids and phenolic acids—also scavenge RCS and free radicals such as reactive oxygen species (ROS), which both demonstrate a prominent role in the pathogenesis of endothelial dysfunction related to diabetes mellitus [22,23]

However, although the therapeutic properties of goat’s rue have long been recognized and appreciated in traditional medicine, the study of the phytochemical profile of the *G. officinalis* herb has been barely addressed. Previous studies on the composition of this species were focused on seed phytochemistry, mainly concerning amino compounds [24]. The chemical constitution of its polyphenolic fraction has received much less attention.

Therefore, the aim of the study was to determine the comprehensive phytochemical composition of the *G. officinalis* herb and the quantitative analysis of individual polyphenolic and guanidine compounds. Moreover, based on the obtained phytochemical profile of *Galegae herba*, rutin, chlorogenic acid, and galegine were selected to represent various groups of natural compounds in galega (flavonoids, hydroxycinnamic acids, and guanidines, respectively). Since the *G. officinalis* herb has been used in the treatment of diabetes for centuries, we decided to test this plant material and selected representative compounds for MGO trapping capacity and non-enzymatic antioxidative activity to assess whether they have properties potentially useful in the prevention of diabetes vascular complications.

## 2. Results and Discussion

### 2.1. Chemical Composition of G. officinalis Extracts

The efficacy of the plant extracts is deemed to rely on the characteristics and activity of their complex chemical components; therefore, a comprehensive analysis of the phytochemical composition of *G. officinalis* herb is crucial in order to understand its potentially beneficial biological mechanisms of action. In the current study, the preliminary UHPLC-ESI-MS analysis indicated a higher content of guanidine and polyphenolic compounds in the hot water extract (infusion) of galega than its aq. methanol extracts. Moreover, the separation of the individual compounds (peaks) was distinctly better in the water extract. The water infusion is also the simplest and most popular pharmaceutical form of medicinal plant preparation and administration. For these reasons, the hot water extract from galega herb was used for further quantitative analysis.

**Figure 1:**
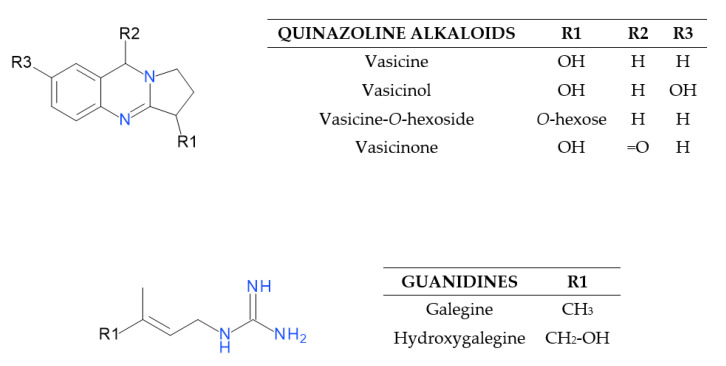
Structures of quinazoline alkaloids and guanidines identified in the *G. officinalis* herb.

The UHPLC-ESI-MS analysis of *G. officinalis* hot water and aq. methanol extracts led to the detection of 39 compounds. Guanidine derivatives and tricyclic quinazoline alkaloids were identified as nitrogen compounds using positive electrospray ionization. In the negative mode, we revealed—to our best knowledge, for the first time—the presence of hydroxycinnamic acid esters (HCAs) with hexaric acid. Other phenolic acids, as well as their sugar derivatives, were also detected. Moreover, various glycosides of flavonols and flavanonols were recognized in the analyzed *G. officinalis* extracts. Identified or tentatively identified constituents along with their *m*/*z* (in negative and/or positive ESI-QqTOF-MS), MS/MS fragments, and maxima of UV-Vis adsorption are presented in Table 1. The chemical structures of the selected identified phytoconstituents are shown in Figure 1. Nevertheless, for most detected flavonoids and HCAs, further spectroscopic analyses should be carried out to clarify their chemical structure.

#### 2.1.1. Characterization of Quinazoline Alkaloids and Guanidines

UHPLC-ESI-MS analysis in positive electrospray ionization mode allowed us to identify or tentatively identify six nitrogen compounds (Figure 1). Peaks 5, 8, 12, and 21 were classified as tricyclic quinazoline alkaloids, whereas 3 and 6 were assigned as guanidine derivatives. Compound 6 (7.5 min) was identified based on comparison with the authentic reference standard as galegine (*m*/*z* 128). Compound 3 (1.49 min) with a pseudomolecular ion at *m*/*z* 144 corresponded to galegine with a hydroxyl group and was identified as hydroxygalegine. Peaks 8 and 5 with retention times of 8.74 and 5.48 min gave [M + H]^+^ ions at *m*/*z* 189 and 205. Compound 5 generated a fragment ion at *m*/*z* 187, which indicated the neutral loss of a water molecule [M − 18 + H]^+^. They were proposed as vasicine (peganine) and hydroxyvasicine (vasicinol), respectively. Their MS/MS fragments were further matched with the literature data [25]. Compound 12 detected at *m*/*z* 351 (12.31 min) was tentatively identified as vasicine-*O*-hexoside. It showed an initial loss of hexose, producing a fragment ion at *m*/*z* 189 [M − 162 + H]^+^ corresponding to vasicine. Compound 21 showed a base peak [M + H]^+^ at *m*/*z* 203 and an MS/MS fragment at *m*/*z* 185, which indicated the loss of water. It was tentatively identified as vasicinone based on fragmentation information and data from the literature [26].

#### 2.1.2. Characterization of Phenolic Acids

**Figure 2:**
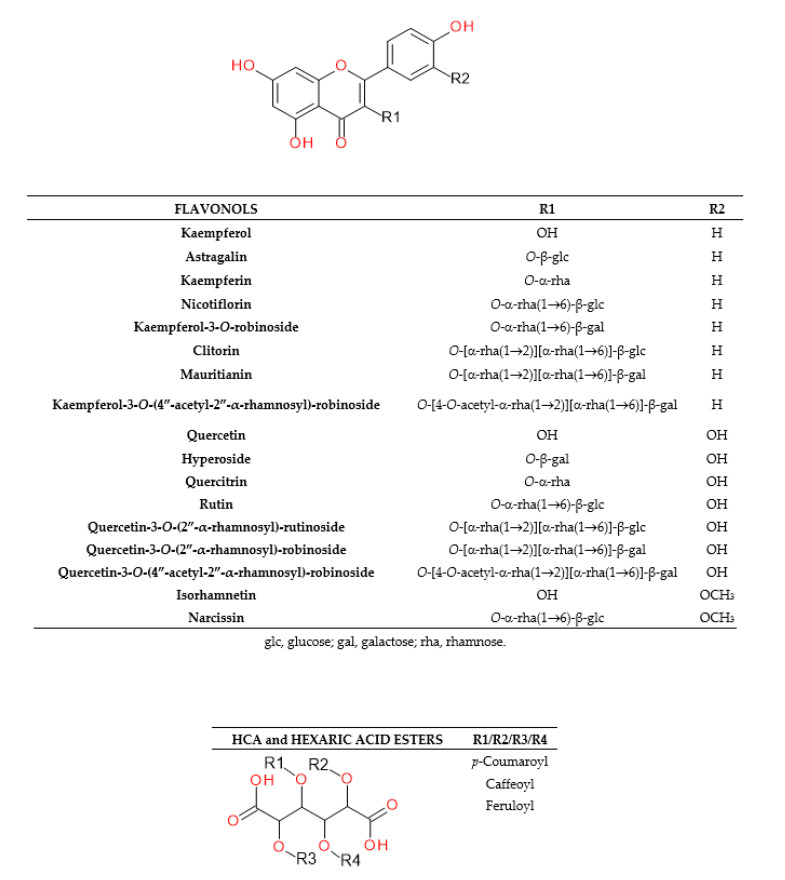
Structures of the flavonols and HCAs identified in the *G. officinalis* herb.

**Figure 3:**
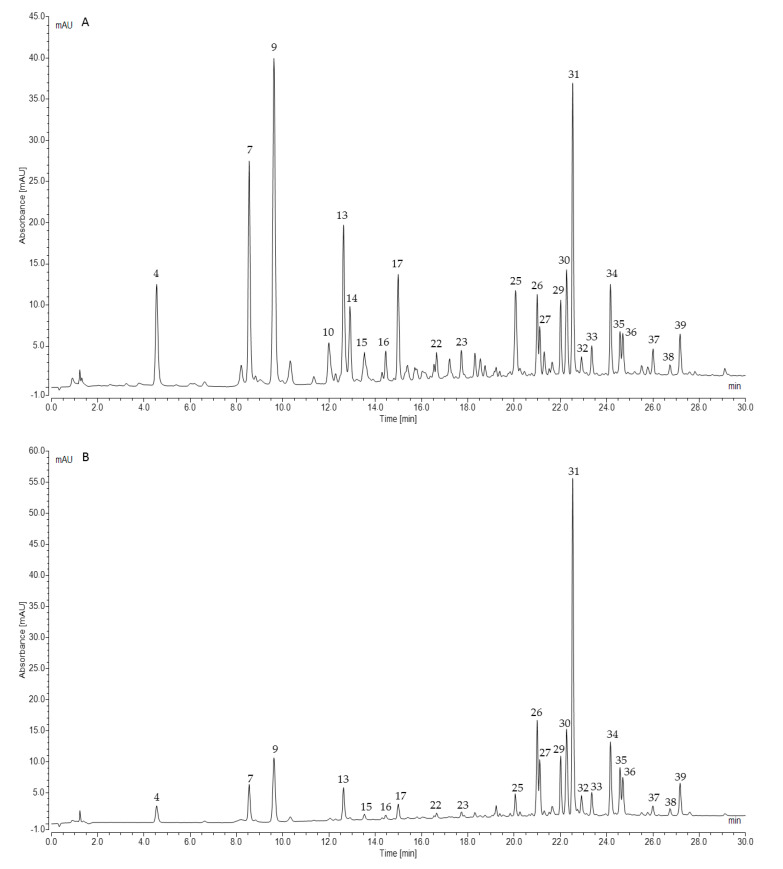
Representative UHPLC chromatograms of *G. officinalis* water infusion at 320 nm (**A**) and 360 nm (**B**).

An analysis of hydroxycinnamic acid (HCAs, t_R_ at 4.32–18.39 min) and hydroxybenzoic acid (HBAs, 11.92 and 15.12 min) derivatives was carried out in negative electrospray ionization mode. Esters of caffeic, ferulic, and *p*-coumaric acids with hexaric acid (aldaric acid) were recognized as predominant HCAs (Figure 2 and Figure 3). Free hexaric acid and its monolactone were also identified as peaks 1 and 2 with *m*/*z* 209 and 191. Four compounds, 4, 7, 9, and 13, with retention times of 4.32, 8.34, 9.49, and 12.43 min, and *m*/*z* 371, were tentatively identified as monocaffeoylhexaric acid isomers, based on the presence of a fragment ion at *m*/*z* 209 which indicates a neutral loss of a caffeoyl moiety [M − 162 − H]^−^. All monocaffeoylhexaric acids also represented MS/MS fragments derived from hexaric acid at *m*/*z* 191, 147, 129, and 111 due to the neutral loss of a water molecule [209 − 18 − H]^−^, CO_2_ and a water molecule [209 − 44 − 18 − H]^−^, CO_2_ and two H_2_O molecules [209 − 44 − 36 − H]^−^, and CO_2_ and three H_2_O molecules [209 − 44 − 54 − H]^−^, respectively, as well as fragments at *m*/*z* 179 and 135, which suggest the occurrence of a caffeic acid residue [CA − H]^−^ and its decarboxylated structure [CA – 44 − H]^−^. Compounds 10, 14, and 20 (t_R_ at 11.84, 12.71, 15.21 min) with *m*/*z* 355 were tentatively identified as three isomers of monocoumaroylhexaric acid (most likely esters of *p*-coumaric acid, pCuA was confirmed as a free acid). All these esters produced fragment ions at *m*/*z* 209, corresponding to a neutral loss of a coumaroyl moiety [M – 146 − H]^−^ and *m*/*z* 163 and 119, which indicated the presence of a coumaric acid residue [CuA − H]^−^ and its decarboxylated form [CuA − 44 − H]^−^. Peaks 15, 16, 17, and 22 (t_R_ at 13.24, 14.3, 14.82, 16.54 min) with pseudomolecular ions of 385 Da were assigned as four monoferuloylhexaric acids. They were tentatively identified based on the occurrence of fragments at *m*/*z* 209 resulting from a neutral loss of a feruloyl moiety [M − 176 − H]^−^. Both monocoumaroylhexaric and monoferuloylhexaric acid isomers have shown further fragmentation patterns similar to the monocaffeoylhexaric acids mentioned above. Compounds 23 and 24 (17.59 and 18.39 min) were identified as chlorogenic and *p*-coumaric acid, respectively, based on comparison with authentic standards. Compounds 11 and 19 (11.92 and 15.12 min) with [M − H]^−^ ions at *m*/*z* 285 and 417 were tentatively identified as protocatechuic acid *O*-pentoside and *O*-dipentoside. They produced a radical aglycone ion [Y_0_ − H]^−•^ at *m*/*z* 152 and 108, as well as an aglycone fragment ion at *m*/*z* 153 and 109 (by the homolytic and heterolytic fission of precursor ions) [27], corresponding to a protocatechuic acid residue after the neutral loss of pentose or dipentose followed by decarboxylation. Similarly, compound 18 (15.1 min) was assigned as coumaric acid *O*-hexoside, exhibiting a deprotonated ion at *m*/*z* 325. It also presented an MS/MS fragment at *m*/*z* 163, which suggests a neutral loss of hexose, and a fragment at *m*/*z* 119, corresponding to coumaric acid after the loss of hexose followed by the loss of CO_2_.

#### 2.1.3. Characterization of Flavonoids

Mono-, di- and triglycosylated flavonols and flavanonols were characterized based on their UV-Vis (200–600 nm) and MS spectra (in the negative and positive ion mode), and, in some cases, by comparison with authentic standards (t_R_). Depending on the structure, the CID of deprotonated flavonoid glycosides produced both radical aglycone and aglycone fragments. In the negative ion mode for flavonols, both homolytic and heterolytic fission was observed under the MS/MS conditions used. In the case of kaempferol glycosides, homolytic fission led to a radical aglycone anion [Y_0_ − H]^−•^ at *m*/*z* 284, while heterolytic fission resulted in an anaglycone fragment [Y_0_]^−^ at *m*/*z* 285. Similar fragmentation behavior was found for quercetin derivatives (*m*/*z* 300 and 301) (**1**). 

The relative abundance of the radical aglycone to the aglycone product ion was found to be dependent on the collision energy as well as the structure of the aglycone and glycone parts. A relative increase in the radical aglycone product ion formation at a higher collision energy was observed by Hvattum and Ekeberg [28], Cuyckens and Claeys [29], and Davis and Brodbelt [30]. For the reference flavonols, including kaempferol-3-*O*-rutinoside (nicotiflorin), kaempferol-7-*O*-neohesperidoside, quercetin-3-*O*-rutinoside (rutin), quercetin-3-*O*-β-glucoside (isoquercitrin), quercetin-3-*O*-β-galactoside (hyperoside), and quercetin-3-*O*-α-rhamnoside (quercitrin), the collision energy of 20 eV resulted primarily in the formation of aglycone fragment [Y_0_]^−^, whereas a collision energy of 40 eV or higher led to the occurrence of the radical aglycone anion [Y_0_ − H]^−•^ [28]. The position of the glycone substitution also affected the fragmentation of the flavonol glycosides. The radical aglycone ions were very abundant for flavonols substituted at C-3. In addition, the ratio [Y_0_ − H]^−•^: [Y_0_]^−^ allows the differentiation between flavonol-3-*O*- and -7-*O*-glycosides and can be used in the identification of unknown compounds [29].

The UHPLC-ESI-MS analysis of flavonoids in the negative electrospray ionization mode led us to the identification or tentative identification of fifteen compounds eluted between 19.98 and 27.13 min (Table 1, Figure 2 and Figure 3). Most of the detected flavonoids were *O*-glycosides of flavonols (kaempferol, quercetin, isorhamnetin). We identified seven kaempferol glycosides (28, 29, 30, 33, 34, 38 and 39), six quercetin glycosides (26, 27, 31, 32, 36, and 37), and only one isorhamnetin derivative (35). Moreover, one flavanonol (taxifolin glycoside, 25) was observed. Glucose, galactose, and rhamnose were connected to aglycones as mono-, di-, or trisaccharides.

Peak 25 (19.98 min) with *m*/*z* 465 was proposed as taxifolin-3-*O*-hexoside, based on the presence of a fragment ion at *m*/*z* 303 [M − 162 − H]^−^, indicating a neutral loss of hexose, and 285 [M − 162 − 18 − H]^−^ after the loss of a water molecule, as well as its characteristic UV-Vis spectrum with λ_max_ at 227 and 289 nm. Compounds 26 and 27 with retention times of 20.49 and 21.06 min and *m*/*z* 755 were proposed as quercetin-3-*O*-dideoxyhexosyl-hexoside isomers. These compounds produced weak MS/MS fragments at *m*/*z* 609 [M − 146 − H]^−^ and base ions at *m*/*z* 300/301 corresponding to quercetin, which indicated the successive loss of deoxyhexose and disaccharide composed of deoxyhexose and hexose units, from the C-3 position of aglycone. The λ_max_ in their UV-Vis spectrum at 254 and 354 nm confirmed that observation. Champavier et al. [31] previously isolated from *G. officinalis* and elucidated the triglycosidic structure of quercetin-3-*O*-[α-rhamnosyl-(1→2)][α-rhamnosyl-(1→6)]-β-glucoside (quercetin-3-*O*-(2″-α-rhamnosyl)-rutinoside). The same compound and its isomer with β-galactose in place of β-glucose were identified by Hirose et al. [31] in quinoa seeds (*Chenopodium quinoa* Willd.). Therefore, 26 and 27 were carefully characterized as quercetin-3-*O*-[α-rhamnosyl-(1→2)][α-rhamnosyl-(1→6)]-β-glucoside or quercetin-3-*O*-[α-rhamnosyl-(1→2)][α-rhamnosyl-(1→6)]-galactoside (quercetin-3-*O*-(2″-α-rhamnosyl)-robinoside). Other quercetin derivatives, compounds 31, 32, and 36 with [M − H]^−^ at 609, 463, and 447,were identified as rutin, hyperoside, and quercitrin based on a comparison with authentic standards and base ions at *m*/*z* 300/301, corresponding to aglycone after the loss of a glycosyl group from C-3.

Compounds 29 and 30 (21.97 and 22.21 min) with *m*/*z* at 739 produced the MS/MS fragment at 284/285 corresponding to kaempferol and suggested the loss of two deoxyhexoses and one hexose from the glycone part [M − 146 − 146 − 162 − H]^−^. The λ_max_ in their UV-Vis spectrum at 265 and 344 nm confirmed the aglycone structure. These compounds were proposed as kaempferol-3-*O*-dideoxyhexosyl-hexoside isomers, probably with a branched triglycoside at C-3 exactly as in 26 and 27. Thus, 29 and 30 were tentatively recognized as kaepferol-3-*O*-[α-rhamnosyl-(1→2)][α-rhamnosyl-(1→6)]-β-galactoside (mauritianin) or kaempferol-3-*O*-[α-rhamnosyl-(1→2)][α-rhamnosyl-(1→6)]-β-glucoside (clitorin). Mauritianin was previously isolated from galega herb by Champavier et al. [31]. Both these triglycosides were also separated from the aerial parts of *Acalypha indica* L. [32] and *Salvadora persica* L. [33]. Two next compounds, 33 and 34 at t_R_ 23.32 and 24.12 min with *m*/*z* 593, showed the same fragmentation patterns. Their MS/MS spectra revealed ions corresponding to kaempferol released after the neutral loss of disaccharide [M − 146 − 162 − H]^−^ composed of deoxyhexose and hexose (probably rhamnose and glucose or galactose). Therefore, they were suggested to be kaempferol-3-*O-*rutinoside (nicotiflorin) after comparison with the authentic standard, and kaempferol-3-*O-*robinoside (kaempferol-3-*O-*robinobioside), respectively. Compounds 28 and 38 (21.61 and 26.69 min) exhibited [M − H]^−^ ions at *m*/*z* 447 and 431, and the same strong fragment related to the base ion at *m*/*z* 285. The loss of 162 Da from the pseudomolecular ion was related to hexose; therefore, compound 28 was assigned by comparison with the reference standard as astragalin. Compound 38 demonstrated the loss of deoxyhexose [M – 146 − H]^−^ and was initially elucidated as kaempferol-3-*O*-α-rhamnoside (kaempferin). Peak 35 (24.53 min) showed a pseudomolecular ion at *m*/*z* 623 and MS/MS fragments with 315 and 300 Da (after the cleavage of the methyl radical). The data suggest that 35 would be identified as isorhamnetin-*O*-deoxyhexosyl-hexoside, and comparison with the authentic standard confirmed identification of isorhamnetin-3-*O*-rutinoside (narcissin). Compound 39 with a pseudomolecular ion at *m*/*z* 781 and a retention time of 27.13 min displayed the MS/MS fragment at 284/285, which suggested the presence of kaempferol aglycone substituted in position C-3. Owing to the absence of pure standards, the literature data were diagnostic. Champavier et al. [34] reported the presence of characteristic acetyl triglycoside in *G. officinalis*. After comparison with data from this study, we identified compound 39 as kaempferol-3-*O*-[4-*O*-acetyl-α-rhamnosyl(1→2)][α-rhamnosyl(1→6)]-β-galactoside (kaempferol-3-*O*-(4″-acetyl-2″-α-rhamnosyl)-robinoside). Peak 37 exhibited a deprotonated molecule at *m*/*z* 797 and produced a fragment ion at *m*/*z* 300/301, which indicated quercetin being aglycon. Based on the above-mentioned study, analogously to compound 39 we tentatively assigned 37 as quercetin-3-*O*-[4-*O*-acetyl-α-rhamnosyl(1→2)][α-rhamnosyl(1→6)]-β-galactoside (quercetin-3-*O*-(4″-acetyl-2″-α-rhamnosyl)-robinoside) or its analog with glucose.

Previous phytochemical research reported that, apart from guanidine derivatives, galega is a rich source of polyphenols, saponins (derivatives of soybean sapogenols, characteristic of the *Fabaceae* family), and alkaloids—mostly quinazoline alkaloids, such as vasicine and vasicinone. Allantoin, medicagol, its methyl ester, and norterpenoid glucoside were isolated from the aerial parts of *G. officinalis* [34]. Terpenes, steroids, tannins, and flavonoids were also found in this plant [35]. The results of the HPLC study by Barchuk et al. [36] revealed the occurrence of 48 phenolic compounds in aq. alcoholic extracts from the galega herb, amongst which only seven polyphenolic compounds were identified: caffeic acid, ferulic acid, cichoric acid, rutin, quercetin, hyperoside, and apigenin. These results are somewhat in agreement with our study. We confirmed the presence of hyperoside and rutin. Quercetin was observed only in the form of glycosides, but caffeic and ferulic acids were observed in the ester forms.

### 2.2. Quantification of Polyphenols and Guanidines

Before the quantitative analysis, the identification of the compounds present in galega extracts was carried out using the UHPLC-ESI-MS method in the negative and positive electrospray ionization modes. The polyphenolic components detected in *G. officinalis* were classified into two main groups based on structural identification: flavonoids and hydroxycinnamic acids (HCAs). Hence, the individual 24 polyphenolic components in the *Galegae herba* water infusion were quantified by using the authentic standards or corresponding standards for calibration for each group (e.g., rutin as an external reference standard for tentatively identified flavonoids and the respective phenolic acid—caffeic, ferulic, or *p*-coumaric acid for HCAs). Flavonoids and HCAs were quantified by the UHPLC-DAD method, whereas guanidines were quantified by UHPLC-ESI-MS using the same chromatographic conditions and a positive ion mode. The results of chromatographic method validation for authentic and corresponding standards, of which the calibration curves exhibited good linearity at 360, 320, and 280 nm (R^2^ = 0.9999) are shown in Table 5 (method section). The results from the quantification are summarized in Table 2. Each individual component was expressed as the mg per 1 g of dried herb ± standard deviation (SD).

Our study revealed that the predominant flavonoid compound presented in the examined batches was rutin, with the concentration of 1.7–3.3 mg/g DW (on average 2.43 mg/g), followed by quercetin-derivative (26), taxifolin-3-*O*-hexoside (25), and mauritianin or clitorin (peaks 29 or 30) with average concentrations of 0.75, 0.68, and 0.65 mg/g DW, respectively. Taxifolin-3-*O*-hexoside was present at a very varied level between the individual batches of the product, and the highest concentration was assessed in Gof1, up to 1.7 mg/g DW. Among the analyzed HCAs, monocaffeoylhexaric acid isomer 3 (peak 9) was the major component, with a concentration of up to 1.64 mg/g DW (on average 1.46 mg/g). The average content of the other isomers of monocaffeoylhexaric acids 1,2,4 (peaks 4, 7, 13) were 0.52, 0.95, and 0.46 mg/g DW, respectively. Monocoumaroylhexaric and monoferuloylhexaric acid isomers were quantified as minor components below 0.45 mg/g DW. The mean flavonoid sum in the analyzed samples was calculated as 6.63 mg/g DW. The average sums of HCAs and all the quantified polyphenols were 5.04 and 11.67 mg/g DW, respectively. The batch assigned as Gof2 had the highest sums of flavonoids and polyphenols, while the lowest values were observed in Gof3.

Since guanidine derivatives cannot be quantified using DAD, we decided to quantify the content of galegine and hydroxygalegine by UHPLC-ESI-MS using the chromatographic conditions described in paragraph 1.4 (method section). Galegine sulfate was used as an external standard for linear regression analysis (Table 5). Galegine was present in Galegae herba at the mean concentration of 6.57 mg/g DW. Its highest content was assessed in Gof2 and was 9.37 mg/g DW, while the lowest was in Gof1 and was 4.27 mg/g DW. The average concentration of hydroxygalegine was several times lower, at −1.51 mg/g DW. The highest concentration of hydroxygalegine was observed in Gof1 (1.98 mg/g DW). The mean sum of guanidines in the analyzed samples was calculated as 8.08 mg/g DW. A study of Oldham et al. [24] indicated that the galegine content varied over the plant tissues and growth stages. The highest level of this compound was observed for reproductive tissues (6.32–8.53 mg/g, on average 7.35 mg/g DW), followed by leaves (2.8–6.22 mg/g, on average 4.25 mg/g DW) and the stem (1.27–1.85 mg/g, on average 1.44 mg/g DW). The examined *G. officinalis* herb consists of flowering aerial parts, which explains the relatively high average concentration of galegine.

Therefore, based on the above results, the average daily dose of galega (4 g) recommended for therapeutic purposes contains ~32 mg of guanidines and 47 mg of polyphenols, including about 26 mg of flavonoids and 20 mg of HCAs.

### 2.3. In Vitro Studies

Many studies suggest that late diabetic complications arise from the reactive carbonyl species’ (especially by MGO) induced formation and generation of advanced glycation end products (AGEs) [37,38]. Glycation is usually accompanied by the process of protein oxidation, and when they occur simultaneously, interacting and intensifying each other’s adverse effects, they are referred to as glycoxidation processes. In addition to AGEs, analogously advanced oxidation protein products (AOPPs) are created [23,39]. There is no doubt a link between oxidative stress, reactive carbonyl species generation, and the development of diabetic complications. Compounds from various chemical groups may reduce both the in vitro and in vivo non-enzymatic glycation and oxidation of proteins by, among other means, trapping RCS or by acting as scavengers of ROS. Therefore, the MGO trapping capacity and antioxidant activity may contribute to limiting the damage from glycation and oxidation reactions and to complementing existing therapy for the treatment of T2D and its vascular complications.

#### 2.3.1. Non-Enzymatic Antioxidant Activity

The DPPH and ABTS assays are frequently used methods for the evaluation of the antioxidant capacities of natural products; both are spectrophotometric techniques based on the quenching of stable radicals [40,41,42]. In the current study, hot water and aq. methanol extracts of *Galegae herba*, and individual standard compounds, were assessed for antioxidant activity using the above-mentioned methods. Table 3 shows the values of antioxidant activity expressed as the percent of inhibition and the concentration required for a 50% reduction in the radicals (IC50, µg/mL; µM) obtained for the tested samples. Standard compounds were selected based on the phytochemical study—rutin, chlorogenic acid, and galegine sulfate were used as model compounds from three different chemical groups (flavonoids, HCA esters, and guanidines). Quercetin was chosen due to the well-known antiradical and antioxidative action, and metformin hydrochloride in order to verify whether the compound with a galegine-like structure, used as the drug of choice in the treatment of T2D, possesses similar properties. The antiradical activity of the extracts and selected substances was compared to the effects of gallic acid (DPPH) and Trolox (ABTS). The IC50 values of the galega extracts were calculated from the mean sum of the polyphenols quantified in *G. officinalis*.

In the current study, both the *G. officinalis* hot water and aq. methanol (1:1) extracts were effective in scavenging DPPH and ABTS radicals, and even more productive than rutin, chlorogenic acid, and trolox. The percentage inhibition of radicals was concentration-dependent and ranged from 3.14% to 75.48% in the DPPH assay and from 10.92% to 87.76% in the ABTS assay for the water infusion, and from 6.78% to 77.16% in DPPH and from 13.57% to 87.85% in ABTS for the aq. methanol extract (both diluted 1–16×). The obtained results are comparable with the previous report by Shymanska et al. [43] of the antioxidant activity of *G. officinalis* leaves, expressed as values of percentage DPPH radical inhibition in the range of 70.4% to 79.7% for methanolic and 54.6% to 66.8% for aqueous extracts. 

The IC50 values of the tested hot water and aq. methanol extracts (calculated for the mean sum of galega polyphenols) were almost identical −12.97 and 11.72 µg/mL in the DPPH method and 1.06 and 0.94 µg/mL in the ABTS assay. The IC50 determined for quercetin in both methods was at a similar level (8.49 and 1.25 µg/mL), which may suggest that the *Galegae herba* antioxidant activity is dependent mainly on the presence of quercetin derivatives. Galegine sulfate showed the lowest inhibitory effect (up 9.49% in ABTS for 9.9 μg/mL), and metformin hydrochloride did not show any antiradical effect under the conditions of the experiment. The IC50 value of the gallic acid standard used as a positive control in the DPPH assay was 2.93 µg/mL (17.25 μM), and trolox, being a positive control in the ABTS test, showed IC50 at 1.48 µg/mL (5.9 μM). 

Taking into account the IC50 values expressed in micromolar concentration, the antiradical activity for the individual compounds is arranged in the following order for the DPPH test: gallic acid > quercetin > rutin > chlorogenic acid >>> galegine sulfate. For the ABTS test, the activity is as follows: quercetin > trolox > rutin > chlorogenic acid >>> galegine sulfate.

Though metformin showed no antiradical action related to electron transfer (DPPH, ABTS assays), some studies report their chelating properties, which may inhibit the metal-catalyzed oxidation reactions that form AGEs [44]. The values of % inhibition calculated for both hot water and methanol extracts (at the concentration of 18 mg/mL in DPPH and 2 mg/mL in ABTS) showed a higher inhibitory activity than rutin and chlorogenic acid but lower than quercetin. This may suggest that the antiradical activity of *G. officinalis* is based not only on polyphenolic compounds. The phytochemical analysis of galega extracts indicated the presence of tricyclic quinazoline alkaloids, which, according to recent research, possess a strong antiradical activity [45] and may contribute to the antioxidative potential of *Galegae herba*. Synergism between the identified components of the test extracts can also be assumed.

#### 2.3.2. Methylglyoxal Trapping Capacity

The MGO trapping test was carried out under the simulated physiological conditions for the freshly prepared hot water extract of *Galegae herba* and standards selected to represent different chemical groups of compounds in the analyzed plant material (rutin, chlorogenic acid and galegine sulfate). As a positive control, quercetin and metformin hydrochloride were used [38,39]. The reaction products were further analyzed with UHPLC-ESI-MS to detect the structure modification of compounds with an MGO trapping potential. Pseudomolecular ions higher by 72 Da in the case of mono-MGO adducts and by 144 Da for di-MGO adducts were searched using the Extract Ion Chromatogram (EIC) function. Phenolic compounds were tested in the negative ion mode, and the guanidine derivatives in the positive mode. Table 4 contains test results for individual compounds and for *G. officinalis* water infusion. Generally, the in vitro study revealed that *G. officinalis* showed MGO trapping activity—some of its components were observed to form adducts with methylglyoxal. Among the tested compounds, rutin, quercetin, galegine sulfate, and metformin hydrochloride exhibited the ability to trap MGO, but trapping activity was not observed for chlorogenic acid. Methylglyoxal reacted with components of *Galegae herba* water infusion—rutin, galegine, and hydroxygalegine. After a 1 h of incubation of the extract with MGO, there were observed two product peaks in positive and one product peak in negative UHPLC-ESI-MS chromatograms. In positive mode, the first peak appeared at 0.98 min with the pseudomolecular ion [M + H]^+^ at *m*/*z* 216, and the second at 2.61 min with *m*/*z* 200, as well as their fragment ions at *m*/*z* 144 and 128 [M – 72 + H]^+^, respectively indicating the loss of one MGO molecule (−72 Da) by MS/MS fission. This suggested that the products were mono-MGO conjugates of hydroxygalegine and galegine. In the negative mode, the product peak observed at 8.84 min characterized the pseudomolecular ion at *m*/*z* 681 [M − H]^−^ and corresponded to the molecule of the mono-MGO adduct of rutin. The peak was 72 mass units higher than that of rutin (*m*/*z* 609).

Moreover, an additional analysis (using EIC) allowed us to observe several slight signals of deprotonated molecules [M − H]^−^ at *m*/*z* 537.1852 and 537.1832 (t_R_ 8.38 and 8.41 min); 827.1950 and 827.1821 (t_R_ 7.93 and 8.18 min); and 665.1476, 665.1490, and 665.1513 (t_R_ 8.97,9.04 and 9.10 min), which corresponded to the adducts of other flavonoids identified in *G. officinalis*, such as taxifolin-3-*O*-hexoside, quercetin-derivatives 26 or 27, and kaempferol-derivatives 33 or 34, respectively (increased by 72 Da). This suggests the presence of their mono-MGO adducts in the extract. The same method allowed us to identify three galegine mono-MGO adducts (t_R_ 1.41, 2.28, and 2.61 min). The first adduct was dominating, while the others were several times smaller. For hydroxygalegine, only one mono-adduct was observed. A similar phenomenon was noted for standards used in the analysis of model mixtures. Rutin, when tested individually, was observed to form three methylglyoxal mono-adducts with [M − H]^−^ at *m*/*z* 681.1682, 681.1695, 681.1683 (t_R_ 8.54, 8.7, 8.82 min), and three di-adducts at *m*/*z* 753.1892, 753.1890, 753.1885 (t_R_ 7.97, 8.24, 8.45 min). Similarly, quercetin was able not only to form mono-adducts (t_R_ 10.93 and 11.06 min), but also the di-adduct with MGO, as shown in Table 4. A study of Bhuiyan et al. [46] suggests that several isomeric forms and diastereoisomers of flavonoid-methylglyoxal adducts may be formed under reaction conditions. In the tested *G. officinalis* hot water extract, the presence of di-MGO adduct of rutin was not detected, perhaps due to the relatively low concentration of rutin or the occurrence of other trapping molecules. 

Galegine sulfate and metformin hydrochloride formed only mono-MGO adducts (Table 4). We observed three mono-MGO adducts for galegine sulfate (by analogy to galegine detected in extract), which could be an effect of substitution of different positions in the guanidine group or other structural differences between the formed adducts. MGO adducts of chlorogenic acid after 1 h of incubation were not observed. Some research showed that chlorogenic acid can inhibit AGE formation induced by methylglyoxal, which suggests that chlorogenic acid scavenges MGO by a mechanism other than direct trapping [47]. 

**Figure 4:**
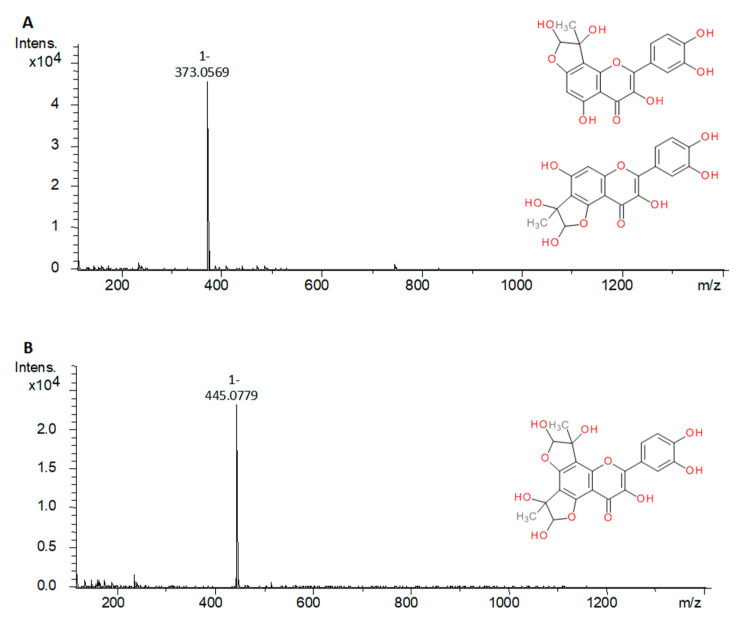

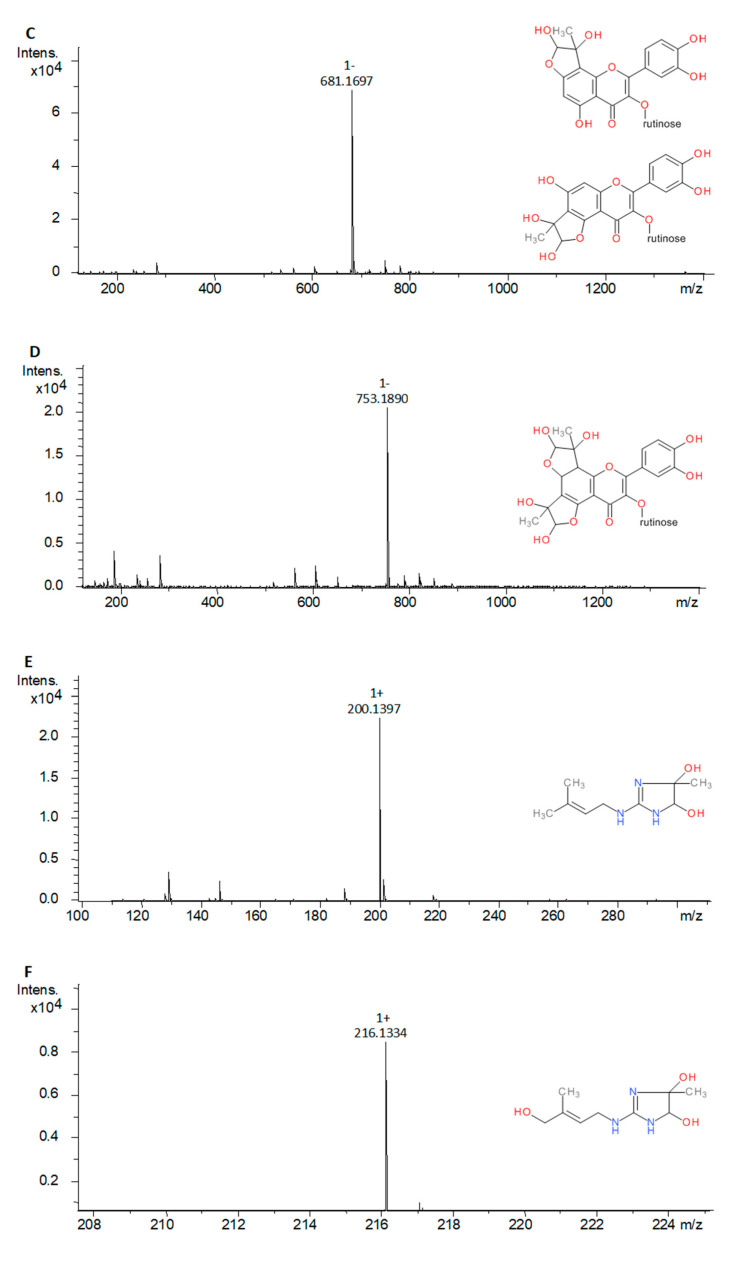

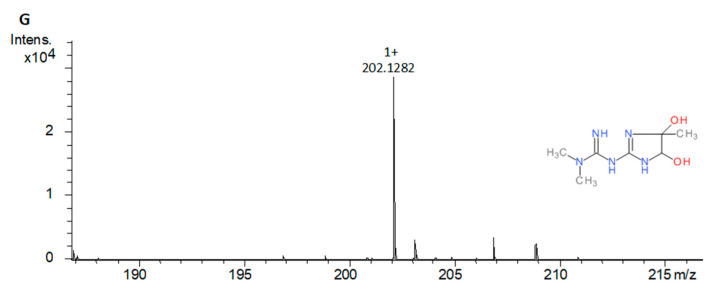
The mass spectra of methylglyoxal adducts and their proposed chemical structure: (**A**), quercetin mono-MGO adduct (373 Da); (**B**), quercetin di-MGO adduct (445 Da); (**C**), rutin mono-MGO adduct (681 Da); (**D**), rutin di-MGO adduct (753 Da); (**E**), galegine mono-MGO adduct (200 Da); (**F**), hydroxygalegine mono-MGO adduct (216 Da); (**G**), metformin mono-MGO adduct (202 Da).

The scientific literature provides information on the formation of mono- and di-MGO adducts with various flavonoids [48,49]. A study by Van den Eynde et al. [50] showed that quercetin traps methylglyoxal effectively enough to significantly reduce its concentration in human plasma. Trapping activity has also been proven for the guanidine derivatives aminoguanidine [51] and metformin [52]; however, to our best knowledge, the ability to trap methylglyoxal by galegine and hydroxygalegine has been demonstrated for the first time in this study. Based on the results of previous structural studies [46,53], we proposed in Figure 4 the chemical structure of the MGO adducts of rutin, galegine, and hydroxygalegine formed in the experimental conditions.

Polyphenols have a beneficial effect on health and can prevent degradative diseases such as T2D, cardiovascular disease, and some cancers through the modulation of several protein functions, as well as antioxidative action and an anti-MGO effect [54]. Flavonols and HCA esters are known as health-promoting and disease-preventing components of many vegetables, fruits, and herbal teas. By contrast, guanidine derivatives are rare plant compounds. Guanidines and quinazoline alkaloids have been identified in several species known for their therapeutic usage but also potential toxicity in livestock [6]. A more recent study by Mooney et al. [55] reported that no toxic effects of galegine were observed in rats at a dose of 600 mg per kilogram body weight for over 28 days. Taking into account the usual daily doses of metformin (up to 3000 mg) in the treatment of T2D patients, the amount of guanidines (32 mg), at the dose of 4 g of *Galegae herba* recommended per day, appears to be safe, and even insufficient to achieve a hypoglycemic effect. Quinazoline alkaloids also possess several interesting pharmacological properties, such as intestinal α-glucosidase and acetylcholine esterase inhibition, as well as anti-inflammatory, antimicrobial, and antioxidative effects [56]. Vasicine and vasicinone were found to be biologically active components of *Adhatoda vasica* (L.) Nees (*Acanthaceae*), a plant used as a herbal medicine for allergen-induced bronchial obstruction and asthma, and as a hepatoprotective and cardioprotective agent [57]. Vasicine was reported to have a protective effect against myocardial infarction [58]. According to the study of Wakhloo et al. [59], vasicine in a dose up to 16 mg (injected intravenous) was well tolerated in humans and showed no undesirable toxic effect in clinical observations. However, the uterus became firm and contracted after long-term vasicine administration, which indicated its oxytocic effect and suggests that it may demonstrate the above-mentioned abortifacient activity [59]. Information available about the safety of quinazoline alkaloids is insufficient, but no adverse effects were reported for *A. vasica* [57].

For these reasons, the use of *G. officinalis* herb in the adjunctive therapy of T2D should be further explored to confirm the underlying mechanism of its action, efficacy, and safety.

## 3. Materials and Methods 

### 3.1. Plant Material

The dried herb of *Galega officinalis* L. (*Galegae herba*) was obtained from the herbal company FLOS (Zakład Konfekcjonowania Ziół FLOS; Mokrsko, Poland) (batches no. 1099, 1108, 1010, assigned as Gof1, Gof2, and Gof3, respectively) certified GMP and ISO 9002. Voucher specimens were deposited in the Herbarium of the Department of Pharmacognosy and Herbal Medicines (Wroclaw Medical University, Wrocław, Poland). Before extractions herb was finely ground in an IKA A11B (IKA Poland Sp. z o.o.; Warsaw, Poland) analytical mill for 5 min.

### 3.2. Chemicals and Standards

The following chemicals were used: methylglyoxal (40% in water), 2,2–diphenyl–1–picrylhydrazyl,2,2-azino-bis-3-ethylbenzothiazoline-6-sulfonic acid, trolox, metformin hydrochloride, 98–100% formic acid, methanol (HPLC grade), acetonitrile (HPLC gradient grade and LC–MS grade), and water (LC–MS grade) were purchased from Merck–Sigma–Aldrich (Sigma–Aldrich Sp. z o.o., Poznań, Poland); NaCl, KCl, Na_2_HPO_4_ and KH_2_PO_4_ (reagent grade) were obtained from Chempur (Piekary Śląskie, Poland); quercetin, quercitrin, astragalin, hyperoside, rutin, nicotiflorin, narcissin, taxifolin, gallic acid, *p*-coumaric acid, ferulic acid, caffeic acid, and chlorogenic acid were from Extrasynthese (Genay Cedex, France); galegine sulfate was purchased from SelectLab (Münster, Germany). Water was glass-distilled and deionized.

The stock solutions of standards (1 mg/mL) were prepared by dissolving 5 mg of a reference compound in 5 mL of methanol. Working standard solutions in the range of 10–400 μg/mL were made by mixing with 50% aq. (aqueous) methanol (*v*/*v*), filtered through hydrophilic Millex Syringe Filters (Durapore 0.22 μm; Millipore, Burlington, MA, USA) and stored at −20 °C.

### 3.3. Preparation of Extracts

A total of 0.2 g of dried and finely powdered plant material was extracted with 10 mL of boiling water for 15 min (infusion) as well with 10 mL of methanol or water-methanol mixture (1:1, 3:7; *v*/*v*) using an ultrasonic bath (Bandelein Sonorex Digital 10P; Bandelin, Berlin, Germany) at 40 °C for 15 min. After 15 min the extracts were passed through a Durapore 0.22 μm filter (Millipore; Burlington, MA, USA) into vials, and the filtrate was analyzed using UHPLC-ESI-MS (Bruker Daltonics; Bremen, Germany) and UHPLC–DAD (Thermo Fisher Scientific; Waltham, MA, USA). The drug extract ratio (DER) was 1:50.

### 3.4. UHPLC-DAD and UHPLC-ESI-MS Analyses

The UHPLC–DAD analyses were conducted on a Thermo Scientific DionexUltiMate 3000 system (Thermo Fisher Scientific; Waltham, MA, USA) equipped with a quaternary pump (LPG-3400D, Thermo Fisher Scientific; Waltham, MA, USA), rapid separation photodiode array detector (DAD–3000) and UltiMate 3000RS autosampler (WPS-3000). The separation of compounds was carried out on a Kinetex C18 column (150 mm × 2.1 mm × 2.6 μm) (Phenomenex; Torrance, CA, USA) and its temperature was maintained at 35 °C using a temperature-controlled column compartment (TCC-3000). The injection volume was 1 µL. The mobile phases used to create the gradient were 0.1% (*v*/*v*) formic acid in water and 0.1% (*v*/*v*) formic acid in acetonitrile as eluents A and B, respectively. The following gradient elution program, at a flow rate of 0.4 mL/min, based on the solvents A and B, was applied: 0–5 min, 100% A; 5–30 min, 100–70% A; 30–32 min, 70–10% A; 32–36 min 10% A. Then, the system returned to the initial setting and was washed with 100% A until the system was stabilized before the next analysis. Spectral measurements were recorded in the wavelength range 200–600 nm, in steps of 2 nm as well as at 220, 254, 280, 320, and 360 nm. Data acquisition was performed with the Chromeleon Chromatography Data System (Thermo Fisher Scientific; Waltham, MA, USA). Amounts of different quantified compounds were calculated as mean values from duplicate UHPLC analyses based on the calibration curves of the corresponding standard compounds or expressed as equivalents of appropriate, related compound (taking into consideration the molar mass differences).

For the UHPLC-ESI-MS analyses, an UHPLC system, set as above, was coupled with Compact ESI-QqTOF-MS (Bruker Daltonics; Bremen, Germany). Nitrogen at 2.0 bar pressure, temperature 210 °C, and flow 0.8 L/min was used as drying and nebulizing gas in the electrospray ionization interface (ESI). Data were acquired in both positive and negative mode. The ion source temperature was set at 100 °C and the capillary voltage was 4500 V (ESI+) or 2200 V (ESI−). The collision energy was 8.0 eV and for MS/MS it was 35 and 40 eV. Sodium formate clusters in concentrations of 10 mM were used for internal calibration. Analyses were run in the same chromatographic conditions as described above. System control and data acquisition were carried out with the Compass Data Analysis software (Bruker Daltonics; Bremen, Germany). The amount of galegine was calculated as the mean value from duplicate analyses based on the calibration curve of galegine sulfate used as an external standard. 

The determination of the methylglyoxal adducts formation was performed using the same UHPLC and QqTOF-MS hardware configuration and mobile phases. The column was thermostatted at 4 ± 1 °C and the injection volume was 1 µL. The following gradient elution program, at a flow rate of 0.3 mL/min, based on the solvents A and B was applied: 0–12 min, 97–65% A; 12–14 min, 65% A; 14–17 min, 65–20% A; 17–19 min 20% A. Then, the system returned to the initial setting and was washed with 97% A until the system was stabilized before the next analysis. The negative and positive-ion polarity mode was applied and other settings were as previously described except capillary voltage, which was set at 5000 V.

### 3.5. Validation of Chromatographic Methods and Quantification

The applied UHPLC methods were validated by the determination of linearity, LOD, and LOQ. The calibration equations for quantified polyphenols and galegine sulfate were assessed at 5 concentration levels, and duplicate injections were performed for each concentration. The values of LOD were established at a signal-to-noise ratio (S/N) of 3 and LOQ were calculated at S/N of 10. The results of the method validation for authentic and corresponding standards, of which the calibration curves exhibited a good linearity (R^2^, 0.9985–0.9999), are shown in Table 5. The average values (mg per 1 g of dried galega herb) and standard deviations (SD) for all the quantified compounds were determined from three independent plant extracts, each in two repetitions.

### 3.6. In Vitro Studies

#### 3.6.1. DPPH Radical Scavenging Assay

The radical scavenging ability against the DPPH radical was measured according to the Blois method with slight modification [60]. In a 96-well microplate, 20 µL of each sample at different concentrations was mixed with 200 µL of 0.3 mM methanolic solution of DPPH. The plate was incubated for 30 min in the dark at ambient temperature and the absorbance was recorded at 517 nm using a Multiskan GO microplate spectrophotometer (Thermo Fisher Scientific; Waltham, MA, USA). Gallic acid was used as a positive control. All the measurements were performed in triplicate. The percentage of DPPH free radical scavenging activity was calculated as follows:

% DPPH scavenge rate = (A0 − A1)/A0 × 100%,(1)

where A0 is the mean absorbance of the control and A1 is the mean absorbance of the extract/standard with DPPH. The IC50 values were calculated using linear regression analysis and used to express the antioxidant capacity.

#### 3.6.2. ABTS Radical Scavenging Assay

The ABTS radical scavenging activity was measured according to the slightly modified method of Chen and Kang [61]. ABTS and potassium persulfate were dissolved in deionized water to final concentrations of 7 and 2.45 mM, respectively. These two solutions were mixed and incubated in the dark at 25 °C for 12 h and subsequently diluted with methanol to reach an absorbance maximum at 734 nm. Next, 200 µL of the obtained reagent was mixed with 2 µL of the tested sample. After incubating at room temperature for 15 min, the absorbance was read at 734 nm using a Multiskan GO microplate spectrophotometer (Thermo Fisher Scientific; Waltham, MA, USA), and Trolox was used as a positive control. All the measurements were performed in triplicate. The ABTS radical scavenging activity was calculated as follows:

% ABTS scavenge rate = (A0 − A1)/A0 × 100%,(2)

where A0 is the mean absorbance of the control and A1 is the mean absorbance of the extract/standard with ABTS. The IC50 values were calculated using linear regression analysis and used to express the antioxidant capacity.

#### 3.6.3. Methylglyoxal Trapping Assay

Methylglyoxal trapping capacity of selected compounds and *G. officinalis* extracts was measured according to the Sang et al. [62] method with slight modification. Briefly, 3 mM methylglyoxal (MGO) was incubated for 1 h with quercetin, rutin, chlorogenic acid, galegine sulfate, metformin hydrochloride (1 mM), and *G. officinalis* hot water extract (1 mL, DER 1:50; equivalent of 20 mg of dried herb) in 100 mM of phosphate buffered saline (PBS; pH 7.4) at 37 °C to equal physiological temperature and shaken at 40 revolutions per minute. The samples were further analyzed using UHPLC-ESI-MS to investigate their ability to form adducts with MGO.

## 4. Conclusions

Previous research proves the hypoglycemic properties of *Galegae herba* extracts but does not draw attention to its potential positive effect on the prevention of vascular complications related to hyperglycemia. Our study proved that *G. officinalis* contains compounds that exhibit both antioxidant activity and the ability to trap methylglyoxal. Both of these properties play an important role in preventing and delaying diabetes complications. 

An in-depth analysis of galega herb revealed the presence not only of guanidines and tricyclic quinazoline alkaloids but also of flavonoid glycosides and HCA esters. The main components in the polyphenolic fraction were flavonols and monocaffeoylhexaric acids. The in vitro tests allowed us to determine which individual *G. officinalis* components show beneficial antioxidative and anti-MGO effects. Flavonols such as rutin and HCAs such as chlorogenic acid exhibited a potent antiradical activity. On the other hand, the ability to trap MGO was noted for guanidines (galegine and hydroxygalegine) and flavonoids (mainly flavonols), whereas the HCA esters and quinazoline alkaloids were ineffective. The UHPLC-ESI-MS method confirmed the presence of mono-MGO adducts of galegine, hydroxygalegine, and metformin, as well as mono-MGO and di-MGO adducts of rutin and quercetin in the reaction mixtures from model tests.

In the light of the results from the phytochemical and in vitro studies, it can be assumed that the polyphenols and guanidines contained in the extracts of *G. officinalis* herb indicate antioxidant and MGO trapping potential, which can be used in the future in the prevention of vascular complications of diabetes. Nevertheless, further research on an in vivo model is necessary.

## Figures and Tables

**Table 1:** UHPLC-ESI-MS data of *G. officinalis* herb components in negative and/or positive ion mode.

Peak No.	t_R_ [min]	λ_max_ [nm]	[M – H]^−^ (*m*/*z*)	Error [ppm]	MS/MS (*m*/*z*)	Identification Proposal
			[M + H]^+^ (*m*/*z*)			
**1**	0.89	200	209.0309	−1.6	165 [M − 44/CO_2_ − H]^−^	Hexaric acid
**2**	1.28	250	191.0203	−1.8	111 [M − 44/CO_2_ − 36/2xH_2_O − H]^−^	Hexaric acid monolactone
**3**	1.49	–	144.1132	−1.2	-	Hydroxygalegine
**4**	4.32	325	371.0618	0.4	209 [M − 162/caffeoyl − H]^−^, 191 [209 − 18/H_2_O − H]^−^, 147 [209 − 18/H_2_O − 44/CO_2_ − H]^−^, 179 [CA − H]^−^, 135 [CA − 44/CO_2_ − H]^−^, 129 [209 − 44/CO_2_ − 36/2xH_2_O − H]^−^, 111 [209 − 44/CO_2_ − 54/3xH_2_O − H]^−^	Monocaffeoylhexaric acid 1
**5**	5.48	–	205.0977	−2.9	187 [M − 18/H_2_O + H]^+^	Vasicinol
**6**	7.5	–	128.1185	−2.2	-	Galegine
**7**	8.34	325	371.0613	1.8	209 [M − 162/caffeoyl − H]^−^, 191 [209 − 18/H_2_O − H]^−^, 147 [209 − 18/H_2_O − 44/CO_2_ − H]^−^, 179 [CA − H]^−^, 135 [CA − 44/CO_2_ − H]^−^, 129[209 − 44/CO_2_ − 36/2xH_2_O − H]^−^, 111 [209 − 44/CO_2_ − 54/3xH_2_O − H]^−^	Monocaffeoylhexaric acid 2
**8**	8.74	206, 285	189.1029	−3.5	171 [M − 18/H_2_O + H]^+^, 144 [M − 18/H_2_O − 27/CHN + H]^+^, 118 [M − 18/H_2_O − 27/CHN − 26/C_2_H_2_ + H]^+^	Vasicine
**9**	9.49	325	371.0621	−0.1	209 [M − 162/caffeoyl − H]^−^, 191 [209 − 18/H_2_O − H]^−^, 147 [209 − 18/H_2_O − 44/CO_2_ − H]^−^, 179 [CA − H]^−^, 135 [CA − 44/CO_2_ − H]^−^, 129[209 − 44/CO_2_ − 36/2xH_2_O − H]^−^, 111 [209 − 44/CO_2_ − 54/3xH_2_O − H]^−^	Monocaffeoylhexaric acid 3
**10**	11.84	313	355.0671	0.9	209 [M − 146/coumaroyl − H]^−^, 191 [209 − 18/H_2_O − H]^−^, 147 [209 − 18/H_2_O − 44/CO_2_ − H]^−^, 163 [CuA − H]^−^, 129 [209 − 44/CO_2_ − 36/2xH_2_O − H]^−^, 119 [CuA − 4/CO_2_ − H]^−^, 111 [209 − 44/CO_2_ − 54/3xH_2_O − H]^−^	Monocoumaroylhexaricacid 1
**11**	11.92	285	285.0617	0.6	152/153 ^a^ [M − 132/pentose − H]^−^, 108/109 ^a^ [PA − 44/CO_2_ − H]^−^	Protocatechuic acid *O*-pentoside
**12**	12.31	208	351.1565	−0.2	189 [M − 162/hexose + H]^+^, 171 [M − 162/hexose − 18/H_2_O + H]^+^	Vasicine-*O*-hexoside
**13**	12.43	325	371.0614	1.6	209 [M − 162/caffeoyl − H]^−^, 191 [209 − 18/H_2_O − H]^−^, 147 [209 − 18/H_2_O − 44/CO_2_ − H]^−^, 179 [CA − H]^−^, 135 [CA − 44/CO_2_ − H]^−^, 129[209 − 44/CO_2_ − 36/2xH_2_O − H]^−^, 111 [209 − 44/CO_2_ − 54/3xH_2_O − H]^−^	Monocaffeoylhexaric acid 4
**14**	12.71	314	355.0671	0.4	209 [M − 146/coumaroyl − H]^−^, 191 [209 − 18/H_2_O − H]^−^, 147 [209 − 18/H_2_O − 44/CO_2_ − H]^−^, 163 [CuA–H]^−^, 129 [209 − 44/CO_2_ − 36/2xH_2_O − H]^−^, 119 [CuA − 44/CO_2_ − H]^−^, 111 [209 − 44/CO_2_ − 54/3xH_2_O − H]^−^	Monocoumaroylhexaricacid 2
**15**	13.24	325	385.0765	0.9	209 [M − 176/feruloyl − H]^−^, 193 [FeA − 191 − H]^−^, 191 [M − 193 − H or 209 − 18/H_2_O − H]^−^, 147 [209 − 18/H_2_O − 44/CO_2_ − H]^−^, 111 [209 − 44/CO_2_ − 54/3xH_2_O − H]^−^	Monoferuloylhexaric acid 1
**16**	14.3	325	385.0775	1.0	209 [M − 176/feruloyl − H]^−^, 193 [FeA − H]^−^, 191 [M − 193 − H or 209 − 18/H_2_O − H]^−^, 147 [209 − 18/H_2_O − 44/CO_2_ − H]^−^, 111 [209 − 44/CO_2_ − 54/3xH_2_O − H]^−^	Monoferuloylhexaric acid 2
**17**	14.82	325	385. 0773	0.7	209 [M − 176/feruloyl − H]^−^, 193 [FeA − H]^−^, 191 [M − 193 − H or 209 − 18/H_2_O − H]^−^, 149 [194v − 44/CO_2_ − H]^−^, 147 [209 − 18/H_2_O − 44/CO_2_ − H]^−^, 111 [209 − 44/CO_2_ − 54/3xH_2_O − H]^−^	Monoferuloylhexaric acid 3
**18**	15.1	315	325.0922	1.5	163 [M − 162/hexose − H]^−^, 119 [CuA − 44/CO_2_ − H]^−^	Coumaric acid *O*-hexoside
**19**	15.12	285	417.1050	−1.6	152/153 ^a^ [M − 264/2 pentose − H]^−^, 108/109 ^a^ [PA − 44/CO_2_ − H]^−^	Protocatechuic acid *O*-di-pentoside
**20**	15.21	312	355.0665	1.2	209 [M − 146/coumaroyl − H]^−^, 191 [209 − 146–18/H_2_O − H]^−^, 147 [209 − 18 − 44/CO_2_ − H]^−^, 163 [CuA − H]^−^, 129 [209 − 44/CO_2_ − 36/2xH_2_O − H]^−^, 119 [CuA − 44/CO_2_ − H]^−^, 111 [209 − 44/CO_2_ − 54/3xH_2_O − H]^−^	Monocoumaroylhexaricacid 3
**21**	15.58	211	203.0825	−4.2	185 [M–18/H_2_O+H]^+^	Vasicinone
**22**	16.54	325	385.0753	1.3	209 [M − 176/feruloyl − H]^−^, 193 [385 − 191 − H]^−^, 191 [M − 193 − H]^−^, 147 [191 − 44/CO_2_ − H]^−^, 111 [209 − 44/CO_2_ − 54/3xH_2_O − H]^−^	Monoferuloylhexaric acid 4
**23**	17.59	325	353.0512	1.8	191 [M − 162/caffeoyl − H]^−^, 179 [CA − H]^−^, 111 [QA − 44/CO_2_ − 36/2xH_2_O − H]^−^	Chlorogenicacid ^S^
**24**	18.39	312	163.0401	−0.1	119 [M − 44/CO_2_ − H]^−^	*p*-Coumaricacid ^S^
**25**	19.98	290	465.1030	1.8	303 [M − 162/hexose − H]^−^, 285 [M − 162/hexose − 18/H_2_O − H]^−^	Taxifolin-3-*O-*hexoside
**26**	20.94	254, 354	755.2036	1.2	609 [M − 146/deoxyhexose − H]^−^, 300/301 ^a^ [M − 2 × 146/dideoxyhexose − 162/hexose − H]^−^	Quercetin-3-*O*-dideoxyhexosyl– hexoside 1
			757.2224	1.3	611[M − 146/deoxyhexose + H]^+^, 303 [M − 2 × 146/dideoxyhexose − 162/Hexose + H]^+^	
**27**	21.06	254, 354	755.2038	0.4	609 [M − 146/deoxyhexose − H]^−^, 300/301 ^a^ [M – 2 × 146/dideoxyhexose – 162/Hexose – H]^−^	Quercetin-3-*O*-dideoxyhexosyl- hexoside 2
			757.2215	0.4	465 [M–2 × 146/dideoxyhexose + H]+, 303 [M – 2 × 146/dideoxyhexose – 162/Hexose + H]^+^	
**28**	21.61	265, 344	447.0942	−0.7	285 [M − 162/glucose – H]^−^	Kaempferol-3-*O*-glucoside (astragalin ^S^)
**29**	21.97	265, 344	739.2088	0.5	284/285 ^a^ [M – 2 × 146/dideoxyhexose − 162/hexose–H]^−^	Kaempferol-3-*O*-dideoxyhexosyl- hexoside 1
**30**	22.21	265, 344	739.2083	1.0	284/285 ^a^ [M – 2 × 146/dideoxyhexose − 162/hexose − H]^−^	Kaempferol-3-*O*-dideoxyhexosyl- hexoside 2
**31**	22.47	255, 353	609.1416	−0.2	300/301 ^a^ [M − 308/rutinose − H]^−^	Quercetin-3-*O*-rutinoside (rutin ^S^)
			611.1634	0,2	303 [M − 308/rutinose + H]^+^	
**32**	22.89	255, 353	463.0870	1.8	300/301 ^a^ [M − 162/galactose − H]^−^	Quercetin-3-*O*-galactoside (hyperoside ^S^)
			465.1037	−1.7	303 [M − 162 + H/galactose]^+^	
**33**	23.32	263, 368	593.1495	3.1	284/285 ^a^ [M − 308/rutinose − H]^−^	Kaempferol-3-*O*-deoxyhexosyl- hexoside 1 (nicotiflorin ^S^)
			595.1681	−3.4	287 [M − 308/rutinose + H]^+^	
**34**	24.12	263, 368	593.1505	1.0	284/285 ^a^ [M − 308/deoxyhexose-hexose − H]^−^	Kaempferol-3-*O*-deoxyhexosyl- hexoside 2
			595.1683	−3.8	287 [M − 308/deoxyhexose-hexose + H]^+^	
**35**	24.53	255, 353	623.1608	2.7	315 [M − 308/rutinose − H]^−^, 300 [M − 308/rutinose − 15/Me^•^ − H]^−•^	Isorhamnetin-3-*O*-rutinoside (narcissin ^S^)
			625.1787	−4.0	317 [M − 308/rutinose + H]^+^, 300 [M − 308/rutinose − 15/Me^•^ + H]^+•^	
**36**	24.66	254, 348	447.0924	2.9	300/301 ^a^ [M − 146/rhamnose − H]^−^	Quercetrin-3-*O*-rhamnoside (quercitrin ^S^)
**37**	25.96	255, 353	797.2135	2.5	300/301 ^a^ [M – 2 × 146/dideoxyhexose − 162/hexose − 42/acetyl − H]^−^	Quercetin-3-*O*-acetyl- dideoxyhexosyl-hexoside
**38**	26.69	263, 368	431.0973	2.2	284/285 ^a^ [M − 146/deoxyhexose − H]^−^	Kaempferol-3-*O*-deoxyhexoside
**39**	27.13	264, 368	781.2192	−0.1	284/285 ^a^ [M – 2 × 146/dideoxyhexose − 162/hexose − 42/acetyl − H]^−^	Kaempferol-3-*O*-acetyl- dideoxyhexosyl-hexoside
			783.2373	−2.7	287 [M – 2 × 146/dideoxyhexose − 162/hexose − 42/acetyl + H]^+^	

t_R_, retention time; λ_max_, absorbance maximum in UV-Vis spectrum; ^a^ [Y0 − H]^−•^/[Y0]^−^; ^S^ reference standard; CA, caffeic acid; CuA, coumaric acid; FeA, ferulic acid; PA, protocatechuic acid; QA, quinic acid.

**Table 2:** Quantification of polyphenolics and guanidines in water infusions (drug extract ratio, 1:50) from three different batches of *G. officinalis* (Gof1-Gof3), expressed as mg per 1 g of dried plant material.

Compound	t_R_ [min]	Gof1	Gof2	Gof3	Average
		Content [mg/g] of DW			
**Flavonoids**					
Taxifolin-3-*O*-hexoside (**25**) ^a^	19.98	1.67 ± 0.20	0.26 ± 0.01	0.03 ± 0.00	0.68 ± 0.65
Quercetin-derivative (**26**) ^b^	20.94	0.59 ± 0.01	0.91 ± 0.02	0.74 ± 0.03	0.75 ± 0.13
Quercetin-derivative (**27**) ^b^	21.06	0.32 ± 0.01	0.47 ± 0.01	0.32 ± 0.01	0.37 ± 0.07
Clitorin (**29**) ^b^	21.97	0.32 ± 0.01	0.47 ± 0.01	0.28 ± 0.02	0.35 ± 0.08
Mauritianin (**30**) ^b^	22.21	0.67 ± 0.01	0.82 ± 0.02	0.47 ± 0.01	0.65 ± 0.15
Rutin (**31**)	22.47	1.70 ± 0.04	3.31 ± 0.07	2.17 ± 0.08	2.43 ± 0.69
Hyperoside (**32**)	22.89	0.08 ± 0.01	0.20 ± 0.01	0.10 ± 0.01	0.13 ± 0.05
Nicotiflorin (**33**) ^b^	23.32	0.14 ±0.01	0.20 ± 0.00	0.13 ± 0.01	0.15 ± 0.03
Kaempferol-3-*O*-robinoside (**34**) ^b^	24.12	0.39 ± 0.19	0.65 ± 0.01	0.44 ± 0.04	0,49 ± 0.06
Narissin (**35**) ^b^	24.53	0.23 ± 0.01	0.35 ± 0.01	0.13 ± 0.01	0.24 ± 0.09
Quercitrin (**36**) ^b^	24.66	0.13 ± 0,01	0.44 ± 0.02	0.02 ± 0.01	0.20 ± 0.19
Kaempferol-derivative (**39**) ^b^	27.13	0.20 ±0.01	0.26 ± 0.01	0.14 ± 0.01	0.19 ± 0.05
Sum of flavonoids		6.44 ± 0.52	8.34 ± 0.20	4.97 ± 0.24	6.63 ± 2.24
**Hydroxycinnamic acids**					
Monocaffeoylhexaric acid isomer 1 (**4**) ^c^	4.32	0.39 ± 0.03	0.57 ± 0.02	0.59 ± 0.03	0.52 ± 0.09
Monocaffeoylhexaric acid isomer 2 (**7**) ^c^	8.34	0.75 ± 0.02	1.05 ± 0.04	1.02 ± 0.03	0.95 ± 0.14
Monocaffeoylhexaric acid isomer 3 (**9**) ^c^	9.49	1.12 ± 0.05	1.58 ± 0.05	1.64 ± 0.22	1.46 ± 0.26
Monocaffeoylhexaric acid isomer 4 (**13**) ^c^	12.43	0.38 ±0.03	0.49 ± 0.02	0.48 ± 0.02	0.46 ± 0.05
Monocoumaroylhexaric acid isomer 1 (**10**) ^d^	11.84	0.20 ± 0.03	0.20 ± 0.01	0.27 ± 0.01	0.22 ± 0.03
Monocoumaroylhexaric acid isomer 2 (**14**) ^d^	12.71	0.35 ± 0.03	0.47 ± 0.04	0.36 ± 0.01	0.39 ± 0.06
Monoferuloylhexaric acid isomer 1 (**15**) ^e^	13.24	0.20 ±0.01	0.25 ± 0.01	0.25 ± 0.01	0.23 ± 0.02
Monoferuloylhexaric acid isomer 2 (**16**) ^e^	14.30	0.12 ± 0.01	0.14 ± 0.01	0.16 ± 0.01	0.14 ± 0.01
Monoferuloylhexaric acid isomer 3 (**17**) ^e^	14.82	0.32 ± 0.02	0.45 ± 0.06	0.47 ± 0.02	0.42 ± 0.07
Monocoumaroylhexaric acid isomer 3 (**20**) ^d^	15.21	0.09 ± 0.01	0.07 ± 0.01	0.12 ± 0.03	0.09 ± 0.02
Monoferuloylhexaric acid isomer 4 (**22**) ^e^	16.54	0.06 ± 0.01	0.06 ± 0.01	0.06 ± 0.01	0.06 ± 0.01
Chlorogenic acid (**23**)	17.59	0.09 ± 0.01	0.12 ± 0.01	0.10 ± 0.01	0.10 ± 0.01
Sum of hydroxycinnamic acids		4.27 ± 0.26	5.45 ± 0.29	5.53 ± 0.41	5.04 ± 0.77
Sum of polyphenols		10.71 ± 0.78	13.79 ± 0.49	10.5 ± 0.65	11.67 ± 3.01
**Guanidines**					
Hydroxygalegine (**3**) ^f^	1.49	1.98 ± 0.03	1.11 ± 0.06	1.95 ± 0.04	1.68 ± 0.12
Galegine (**6**)	7.50	4.28 ± 0.04	9.38 ± 0.40	6.08 ± 0.17	6.58 ± 0.61
Sum of guanidines		6.26 ± 0.07	10.49 ± 0.46	8.03 ± 0.21	8.26 ± 0.73

^a^ quantified as taxifolin; ^b^ as rutin; ^c^ as caffeic acid; ^d^ as *p*-coumaric acid; ^e^ as ferulic acid; ^f^ as galegine; DW, dry weight; all reported values (mg/g) are means of three samples in two measurements (*n* = 3 × 2).

**Table 3:** Antioxidant activity of the *G. officinalis* extracts and selected standard compounds.

Sample	DPPH				ABTS	
	IC50 [µg/mL]	IC50 [μM]	% of Inhibition ^a^	IC50 [µg/mL]	IC50 [μM]	% of Inhibition ^b^
Aq. methanol (1:1) ^c^	11.72 ^d^	-	73.00	0.94 ^d^	-	85.31
Water infusion ^c^	12.97 ^d^	-	68.80	1.06 ^d^	-	82.34
Chlorogenic acid	27.60	77.90	33.08	2.62	7.41	37.00
Rutin	22.29	36.51	42.91	4.07	6.66	31.02
Quercetin	8.49	28.08	91.76	1.25	4.13	87.14
Galegine sulfate	1656.07	13,020.31	0.90	42.52	334.32	0
Metformin hydrochloride	0	0	0	0	0	0
Gallic acid	2.93	17.25	>100	-	-	-
Trolox	-	-	-	1.48	5.90	61.37

Values are mean triplicate (*n* = 3); ^a^ calculated for final concentration 18 µg/mL; ^b^ calculated for final concentration 2 µg/mL; ^c^ extracts from galega herb (DER 1:50); ^d^ calculated from the mean sum of polyphenols.

**Table 4:** Methylglyoxal adducts detected in the reaction mixture of standard compounds and water infusion of *G. officinalis* after 1 h incubation with MGO. Quercetin and metformin hydrochloride were used as a positive control.

Compound	Source	Peak	Mono-MGO Adduct (*m*/*z*)	di-MGO Adduct (*m*/*z*)
Chlorogenic acid	S	-	n.d.	n.d.
Rutin	S	a	681.1682 [M − H]^−^	753.1892 [M − H]^−^
		b	681.1695 [M − H]^−^	753.1890 [M − H]^−^
		c	681.1683 [M − H]^−^	753.1885 [M − H]^−^
	Inf	a	681.1684 [M − H]^−^	n.d.
Quercetin	S	a	373.0569 [M − H]^−^	445.0779 [M − H]^−^
		b	373.0564 [M − H]^−^	n.d.
Galegine sulfate	S	a	200.1367 [M + H]^+^	n.d.
		b	200.1364 [M + H]^+^	n.d.
		c	200.1364 [M + H]^+^	n.d.
Galegine	Inf	a	200.1397 [M + H]^+^	n.d.
		b	200.1386 [M + H]^+^	n.d.
		c	200.1388 [M + H]^+^	n.d.
Hydroxygalegine	Inf	a	216.1334 [M + H]^+^	n.d.
Metformin hydrochloride	S	a	202.1282 [M + H]^+^	n.d.

S: standard compound; Inf, water infusion; n.d.: not detected.

**Table 5:** Validation parameters of the standard compounds for UHPLC-DAD and UHPLC-ESI-MS analysis.

Compound	Method	Λ [nm]	Linear Equation	R^2^	Range [µg/mL]	LOD [µg/mL]	LOQ [µg/mL]
Chlorogenic acid	UHPLC-DAD	320	y = 0.0051x – 0.0009	0.9999	10–250	0.16	0.50
Caffeic acid	UHPLC-DAD	320	y = 0.00301x + 0.00047	0.9999	10–250	0.56	1.87
*p*–Coumaric acid	UHPLC-DAD	320	y = 0.00250x − 0.00003	0.9999	10–250	0.57	1.90
Ferulic acid	UHPLC-DAD	320	y = 0.00321x + 0.00018	0.9999	10–250	0.48	1.61
Hyperoside	UHPLC-DAD	360	y = 0.00612x + 0.00007	0.9999	10–400	0.11	0.36
Rutin	UHPLC-DAD	360	Y = 0.0097x − 0.000007	0.9999	10–400	0.16	0.50
Taxifolin	UHPLC-DAD	280	y = 0.02357x + 0.00163	0.9999	10–400	0.08	0.28
Galegine sulfate	UHPLC-ESI-MS	[M + H]^+^	y = 1.00029x − 0.00008	0.9985	10–400	0.01	0.03

λ, wavelength; y = ax + b, y − peak area; R^2^, coefficient of determination; LOD, limit of detection; LOQ, limit of quantitation; *n* = 2 × 5.

## Abbreviations

T2D	type 2 diabetes mellitus
MGO	methylglyoxal
*G. officinalis*	*Galega officinalis*
t_R_	retention time
λ_max_	absorbance maximum in UV-Vis spectrum
ROS	reactive oxygen species
RCS	reactive carbonyl species
AGEs	advanced glycation end products
AOPPs	advanced oxidation end products
UHPLC	ultra high-performance liquid chromatography
DAD	diode array detector
ESI-MS	electrospray ionization mass spectrometry
ABTS	2,2′-azino-bis(3-ethylbenzthiazoline-6-sulfonic acid)
DPPH	2,2-diphenyl-1-(2,4,6-trinitrophenyl)hydrazyl
HCAs	hydroxycinnamic acids
IC50	the half maximal inhibitory concentration
EIC	extracted ion chromatogram
DER	drug extract ratio
